# Structures of *rac*-2,4:3,5-di­methyl­ene xylitol ­derivatives

**DOI:** 10.1107/S2056989023006497

**Published:** 2023-08-04

**Authors:** Michael Satlow, Paul G. Williard

**Affiliations:** a Program in Judaic Studies-Box 1826, Brown University, Providence, Rhode Island 02912, USA; b Department of Chemistry-Box H, Brown University, Providence, Rhode Island 02912, USA; University of Kentucky, USA

**Keywords:** xylitol, pentose, *cis*-1,3,5,7-tetra­oxadeca­lin, *cis*-deca­lin conformation, crystal structure

## Abstract

The structures of three racemic (tetra­hydro-[1,3]dioxino[5,4-*d*][1,3]dioxin-4-yl)methanol derivatives are reported, namely, 4-[(methyl­sulfon­yloxy)meth­yl]-2,4,4a,6,8,8a-hexa­hydro-[1,3]dioxino[5,4-*d*][1,3]dioxine, C_8_H_14_O_7_S, **1**, 4-[(benz­yloxy)meth­yl]-2,4,4a,6,8,8a-hexa­hydro-[1,3]dioxino[5,4-*d*][1,3]dioxine, C_14_H_18_O_5_, **2**, and 4-[(anilinocarbon­yl)meth­yl]-2,4,4a,6,8,8a-hexa­hydro-[1,3]dioxino[5,4-*d*][1,3]dioxine, C_14_H_17_NO_6_, **3**. Mesylate ester **1** at 173 K has triclinic *P*  symmetry and both benzyl ether **2** at 173 K and phenyl urethane **3** have monoclinic *P*2_1_/*c* symmetry. These structures are of inter­est because of the conformation of the *cis*-fused tetra­oxadeca­lin ring system. This *cis*-bi­cyclo­[4.4.0]decane ring system, *i.e. cis*-deca­lin, can undergo conformational equilibration. In the two most stable conformers, both six-membered rings adopt a chair conformation. However, there are significant consequences in these two stable conformers, with heteroatom substitution at the 1,3,5,7-ring positions as described. Only one conformation, denoted as ‘concave’ or ‘inside’, is found in these crystal structures. This is consistent with previously reported structures of the 1,1-geminal dihy­droxy aldehyde and tosyl­ate analogs.

## Abstract 2

The crystal structures of three xylitol derivatives prepared directly from commercially available xylitol by treatment with formalin and acid followed by subsequent derivatization of the primary hydroxyl group of the bis-methyl­ene ketal with mesyl chloride, benzyl bromide or phenyl iso­cyanate are reported.

## 1. Chemical context

Naturally occurring monosaccharides provide an abundant source of inexpensive, often chiral, starting materials for the syntheses of numerous sophisticated natural products, non-natural physiologically active com­pounds, and ligands for stereoselective catalysts (Ferrier, 2003 ▸). Over the past decade or so, a sharply increasing emphasis is seen on the use of these sugars and also on chemical transformations among the various diastereomeric and homologous series of monosaccharides. Despite this flurry of activity, monosaccharide derivatives still provide a rich source of challenging structural and conformational issues due to the anomeric and *gauche* inter­actions associated with the O atoms. 
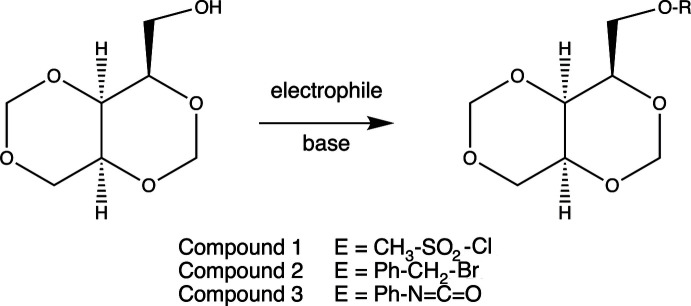


**Figure 1:**
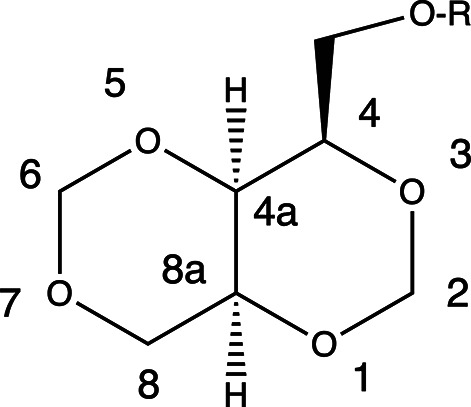
The structures and atom numbering for com­pounds **1**–**3**. For **1**, *R* = SO_2_–CH_3_, for **2**, *R* = CH_2_–Ph, and for **3**, *R* = CO–NH–Ph.

In this article, we describe the crystal structures of three *cis*-fused [4.4.0]bi­cyclo methyl­ene acetals originally derived from the most inexpensive and readily available five-carbon *meso* polyalcohol, *i.e.* xylitol. The chemical structures of these com­pounds are shown in the scheme[Chem scheme1]. The standard chemical numbering for the 1,3,5,7-tetra­oxadeca­lin ring system is shown in Fig. 1 ▸. The atoms in all three crystal structures reported are labeled following this pattern. Compound **1** is a mesylate, with *R* = mesyl (Zarubinskii & Danilov, 1972 ▸), com­pound **2** is a benzyl ether, with *R* = benzyl (Che *et al.*, 2017 ▸), and com­pound **3** is an *N*-phenyl­urethane, with *R* = –CO–NH–Ph. Since xylitol itself is achiral and we carried out no enanti­oselective reactions to prepare chiral derivatives, the structures we report are of racemates and hence centrosymmetric, although it is possible to obtain enanti­omerically pure com­pounds from more com­plicated synthetic routes.

## 2. Structural commentary

**Figure 2:**
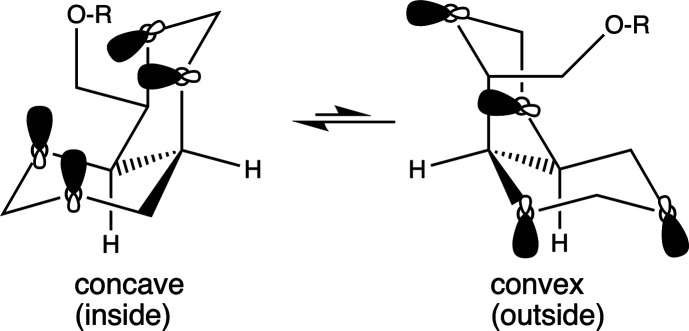
Stable conformations of *cis*-1,3,5,7-tetra­oxa-[4.4.0]bi­cyclo­deca­lin.

The defining characteristic of the *cis*-1,3,5,7-tetra­oxa-[4.4.0]bi­cyclo­deca­lin ring system is depicted in Figs. 2 ▸ and 3 ▸. Fig. 2 ▸ illustrates the two lowest-energy all-chair conformations of this skeleton. The O atoms in these conformers adopt a tetra­hedral geometry and the axial lone pair of electrons on each of these O atoms within the deca­lin ring are depicted. This feature was noted previously (Lemieux & Howard, 1963 ▸; Burkert, 1980 ▸; Taskinen, 2009 ▸) and described in detail in a mini-review summarizing over two decades of chemical work largely from one laboratory (Fuchs, 2013 ▸). Trivial nomenclature has evolved to describe these two conformations as inside/concave or outside/convex. These descriptions derive from the orientation of the axial lone pairs on the ring O atoms relative to the overall shape of the deca­lin ring system. For the com­pletely unsubstituted tetra­oxydeca­lin, it is not immediately obvious which of these two conformers is more stable.

**Figure 3:**
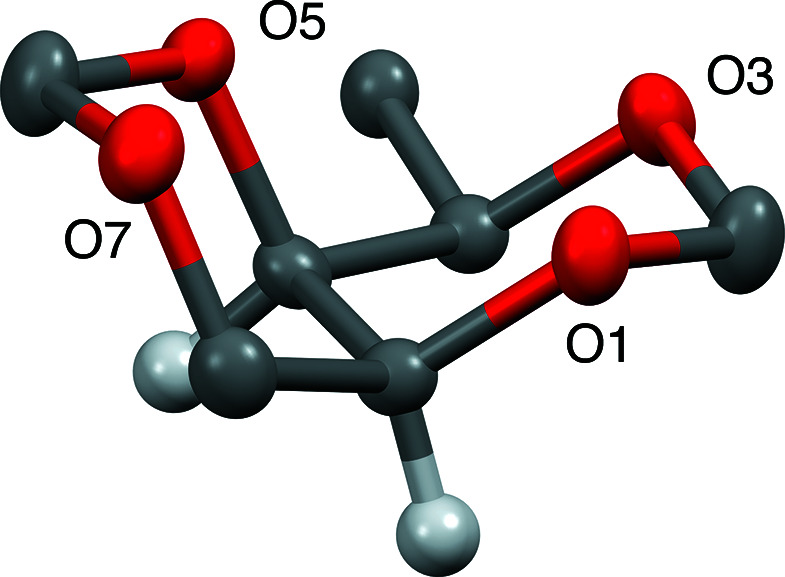
The half-cylinder mol­ecular shape of *cis*-1,3,5,7-tetra­oxa-[4.4.0]bi­cyclo­deca­lin.

Compounds **1**–**3** also incorporate a derivatized hy­droxy­methyl substituent at position C4 that is *trans* to both bridgehead H atoms. Consequently, this substituent must be equatorial in the concave/inside conformer and axial in the convex/outside conformer. Conformational analysis suggests that the concave/inside conformer is favored, as seen in all these crystal structures. Fig. 3 ▸ highlights this overall geometry found in all three crystal structures. The overall shape of this mol­ecule resembles a cylinder that has been cut in half. It is noteworthy that this mol­ecular shape has been examined for its potential to chelate cations as a polydentate ligand (Ganguly & Fuchs, 2001 ▸) and also as a cryptand (Abramson *et al.*, 2003 ▸).

**Figure 4:**
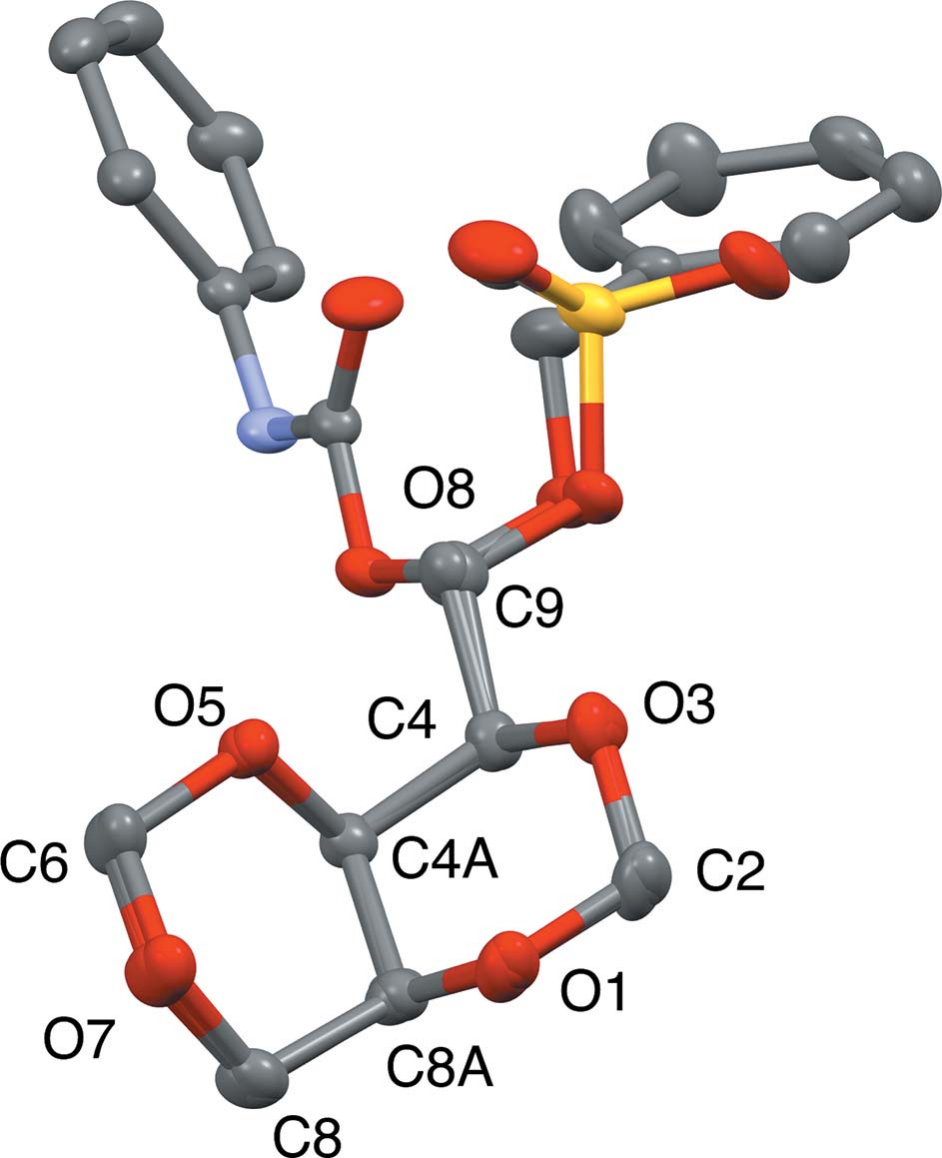
Structural overlay of com­pounds **1**–**3**.

Fig. 4 ▸ is an overlay of all three crystal structures obtained by minimizing the positional differences of the four ring O atoms in all three structures. No significant difference in the geometry of the tetra­oxabicyclic ring in these three structures is discernible. It is noteworthy that a *gauche* conformation is found for the O3—C4—C9—O8 torsion angle, with values of 61.8 (2) and 81.6 (1)° in mesylate **1** and benzyl ether **2**, respectively. However, a relatively anti­periplanar torsion angle of 175.9 (8)° exists in urethane **3**. This is likely the consequence of stabilization by the single inter­molecular hydrogen bond observed in the urethane structure (see below).

**Figure 5:**
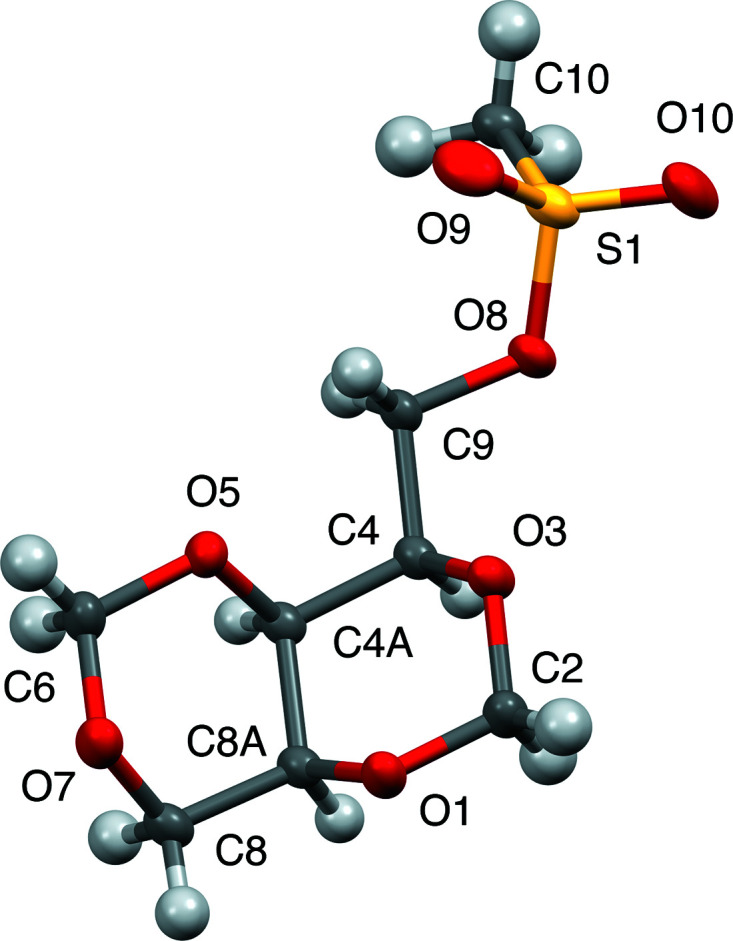
Displacement ellipsoid plot (50% probability) of com­pound **1**.

Figs. 5 ▸–7 ▸ ▸ display the all-atom displacement ellipsoid plots of com­pounds **1**–**3**.

## 3. Supra­molecular features

**Figure 8:**
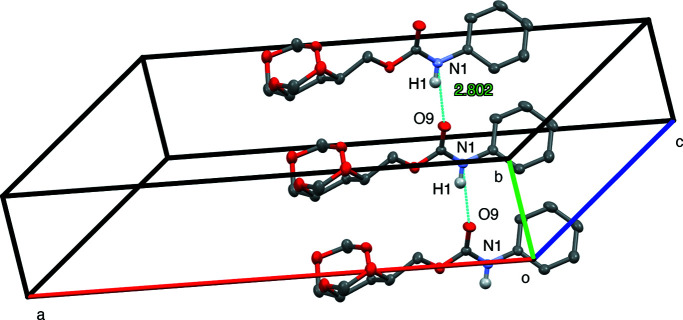
Hydrogen bonding in com­pound **3**.

An intra­molecular N—H⋯O hydrogen bond is observed in phenyl urethane derivative **3** between the –NH substituent and the carbonyl O atom of the urethane functional group. This is described as *D*—H⋯*A* (N1—H1⋯·O9^i^), with N1—H1 = 0.863 (16) Å, H1⋯O9^i^ = 1.969 (16) Å, N1⋯O9^i^ = 2.8025 (13) Å and N1—H1⋯O9^i^ = 161.9 (14)° [symmetry code: (i) *x*, *y* − 1, *z*]. This is shown in Fig. 8 ▸.

No other hydrogen-bond inter­actions are possible in any of the structures, although there are short C—H⋯O inter­actions between the H2*B* atom on a methyl­ene acetal and an adjacent acetal O5^ii^ atom [symmetry code: (ii) *x*, −*y* + 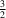 , *z* + 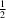 ] in urethane structure **3** that is characteristically seen in all of the structures. This is characterized in Table 1 ▸.

No π-stacking inter­actions of the aromatic rings are observed.

## 4. Database survey

A search of the Cambridge Structural Database (CSD, Version 5.43, update of November 2021; Groom *et al.*, 2016 ▸) for similar structures returned two relevant entries: 2,4:3,5-di-*O*-methyl­ene-1-*p*-toluene­sulfonyl xylitol (CSD refcode HALSAO; Rodier *et al.*, 1993 ▸) and dihy­droxy-2,4:3,5-di­methyl­ene-l-xylose (SIVHUA; Smith *et al.*, 1991 ▸).

## 5. Synthesis and crystallization

Compounds **1** and **2** were prepared and crystallized by the following general procedure. To a solution of racemic 2,4:3,5-di­methyl­ene xylitol (Hann *et al.*, 1944 ▸) in pyridine, 1.1 molar equivalents of either mesyl chloride or benzyl bromide were added and stirred at room temperature until the diacetal dissolved (∼4 h). The resulting reaction mixtures were allowed to stand for 18 h at room temperature and then poured onto crushed ice. Solid crystalline material formed upon slow evaporation of the reaction mixture on sitting in a fume hood overnight. Recrystallization from ethanol pro­duced diffraction-quality crystals. ^1^H and ^13^C NMR spectra of the crystalline samples indicated no discernible impurities and are provided in the supporting information.

Compound **1**, ^13^C{^1^H} NMR (298 K, CDCl_3_, 100.5 MHz): δ 93.07, 92.85, 75.49, 70.13, 69.42, 69.32, 68.30, 37.36.

Compound **2**, ^13^C{^1^H} NMR (298 K, CDCl_3_, 100.5 MHz): δ 137.94, 128.44, 127.85, 127.79, 93.21, 93.16, 77.24, 73.67, 70.63, 70.16, 69.52, 68.49.

For urethane derivative **3**, a solution of racemic 2,4:3,5-di­methyl­ene xylitol, 1.1 molar equivalents of phenyl iso­cyanate, and pyridine was heated to reflux for 2 h protected from atmospheric moisture by a drying tube. On cooling, the derivative precipitated from the solution and was collected by filtration. Recrystallization from acetone yielded diffraction-quality crystals. ^1^H and ^13^C NMR spectra of the crystalline samples indicated no discernible impurities and are provided in the supporting information.

Compound **3**, ^13^C{^1^H} NMR (298 K, *d*_6_-DMSO, 100.5 MHz): δ 153.76, 139.49, 129.21, 122.92, 118.64, 92.56, 92.17, 75.57, 69.98, 69.93, 69.12, 63.86.

## 6. Refinement

Crystal data, data collection and structure refinement details are summarized in Table 2 ▸. H atoms were added automatically using a riding model with *U*_iso_(H) = 1.2*U*_eq_(C). The H atom on N1 in urethane **3** was located in a difference Fourier map and refined freely.

## Supplementary Material

Crystal structure: contains datablock(s) 1, 2, 3, global. DOI: 10.1107/S2056989023006497/pk2693sup1.cif

Structure factors: contains datablock(s) 1. DOI: 10.1107/S2056989023006497/pk26931sup2.hkl

Supporting information file. DOI: 10.1107/S2056989023006497/pk26931sup5.cml

Structure factors: contains datablock(s) 2. DOI: 10.1107/S2056989023006497/pk26932sup3.hkl

Supporting information file. DOI: 10.1107/S2056989023006497/pk26932sup6.cml

Structure factors: contains datablock(s) 3. DOI: 10.1107/S2056989023006497/pk26933sup4.hkl

Supporting information file. DOI: 10.1107/S2056989023006497/pk26933sup7.cml

NMR 1H and 13C-NMR spectra of compounds 1-3. DOI: 10.1107/S2056989023006497/pk2693sup8.pdf

CCDC references: 2284876, 2284877, 2284878

Additional supporting information:  crystallographic information; 3D view; checkCIF report

## Figures and Tables

**Figure 6:**
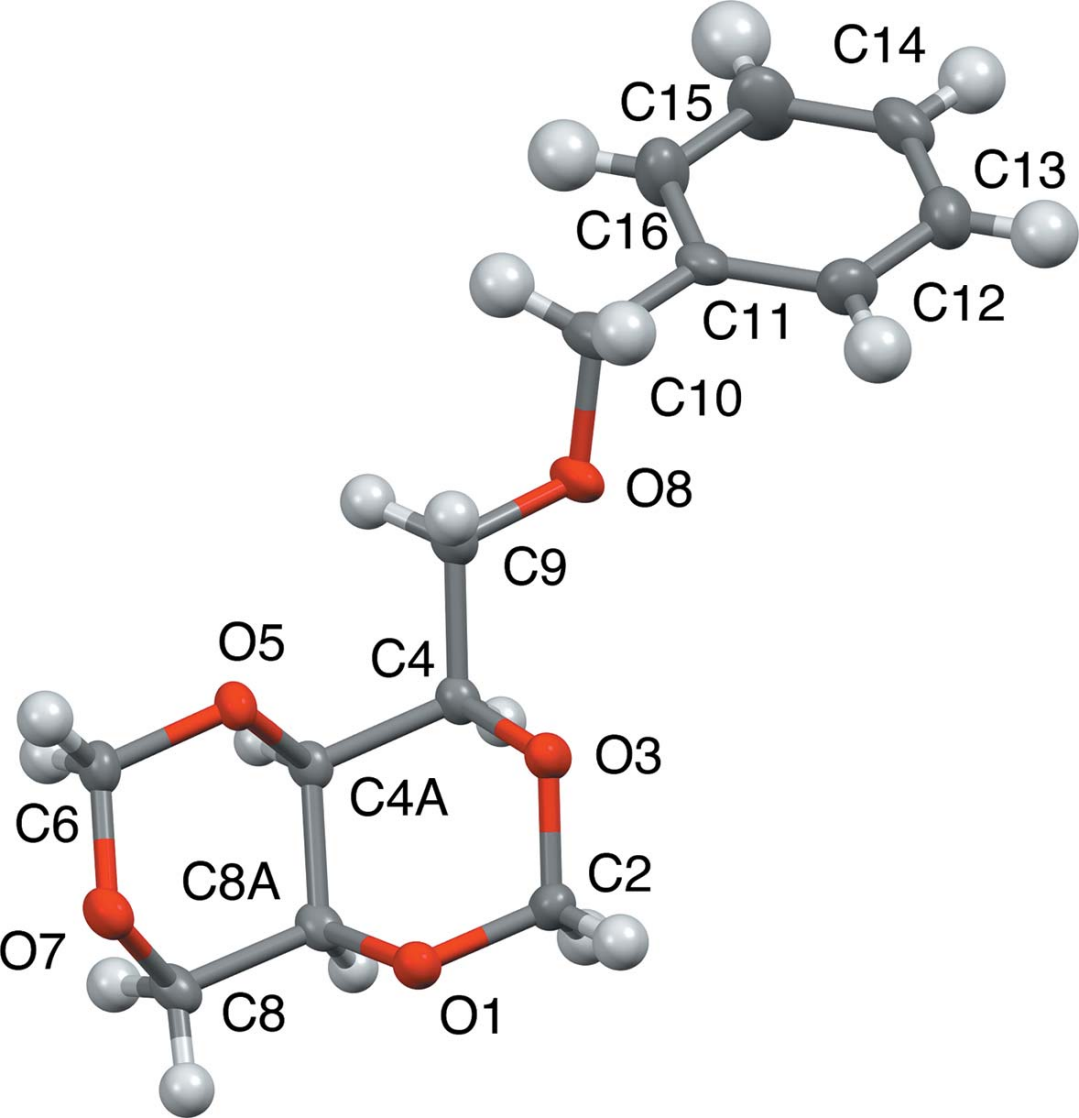
Displacement ellipsoid plot (50% probability) of com­pound **2**.

**Figure 7:**
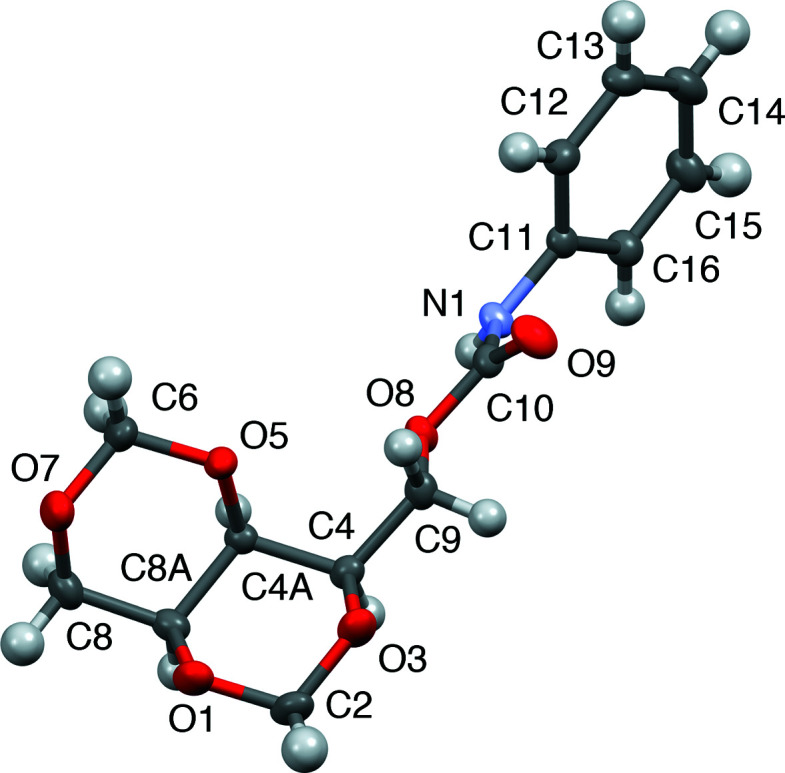
Displacement ellipsoid plot (50% probability) of com­pound **3**.

**Table 1:** Hydrogen-bond geometry (Å, °) for **3** [Chem scheme1]

*D*—H⋯*A*	*D*—H	H⋯*A*	*D*⋯*A*	*D*—H⋯*A*
N1—H1⋯O9^i^	0.863 (16)	1.969 (16)	2.8025 (13)	161.9 (14)
C2—H2*B*⋯O5^ii^	0.99	2.51	3.4515 (15)	159

Symmetry codes: (i) 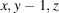 ; (ii) 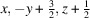 .

**Table 2:** Experimental details Experiments were carried out at 173 K with Mo *K*α radiation. Absorption was corrected for by multi-scan methods (*SADABS*; Krause *et al.*, 2015 ▸).

	**1**	**2**	**3**
Crystal data			
Chemical formula	C_8_H_14_O_7_S	C_14_H_18_O_5_	C_14_H_17_NO_6_
*M*_r_	254.25	266.28	295.28
Crystal system, space group	Triclinic, *P* 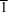	Monoclinic, *P*2_1_/*c*	Monoclinic, *P*2_1_/*c*
*a*, *b*, *c* (Å)	4.7401 (4), 7.3325 (6), 15.9604 (14)	20.5429 (9), 4.4574 (2), 13.9148 (7)	22.909 (2), 4.8973 (5), 12.2331 (14)
α, β, γ (°)	90.019 (3), 93.610 (3), 106.439 (3)	90, 96.651 (2), 90	90, 104.529 (4), 90
*V* (Å^3^)	530.90 (8)	1265.57 (10)	1328.6 (2)
*Z*	2	4	4
μ (mm^−1^)	0.32	0.11	0.12
Crystal size (mm)	0.15 × 0.13 × 0.09	0.20 × 0.10 × 0.08	0.20 × 0.15 × 0.12

Data collection			
Diffractometer	Bruker D8 Quest	Bruker D8 Quest	Bruker D8 Venture Duo
*T*_min_, *T*_max_	0.665, 0.748	0.712, 0.746	0.568, 0.746
No. of measured, independent and observed [*I* > 2σ(*I*)] reflections	15993, 4823, 2290	32635, 3878, 2759	23485, 3040, 2803
*R*_int_	0.084	0.062	0.069
(sin θ/λ)_max_ (Å^−1^)	0.929	0.716	0.650

Refinement			
*R*[*F* ^2^ > 2σ(*F* ^2^)], *wR*(*F* ^2^), *S*	0.070, 0.147, 1.01	0.051, 0.123, 1.04	0.044, 0.107, 1.06
No. of reflections	4823	3878	3040
No. of parameters	146	172	193
H-atom treatment	H-atom parameters constrained	H-atom parameters constrained	H atoms treated by a mixture of independent and constrained refinement
Δρ_max_, Δρ_min_ (e Å^−3^)	0.41, −0.66	0.31, −0.21	0.34, −0.29

Computer programs: *APEX4* and *SAINT* (Bruker, 2022 ▸), *SHELXT2019* (Sheldrick, 2015*a* ▸), *SHELXL2019* (Sheldrick, 2015*b* ▸), *Mercury* (Macrae *et al.*, 2020 ▸), and *publCIF* (Westrip, 2010 ▸).

## supplementary crystallographic information

### 4-[(Methylsulfonyloxy)methyl]-2,4,4a,6,8,8a-hexahydro-[1,3]dioxino[5,4-d][1,3]dioxine (1) Crystal data

**Table:**

C_8_H_14_O_7_S	*Z* = 2
*M**_r_* = 254.25	*F*(000) = 268
Triclinic, *P*1	*D*_x_ = 1.590 Mg m^−^^3^
*a* = 4.7401 (4) Å	Mo *K*α radiation, λ = 0.71073 Å
*b* = 7.3325 (6) Å	Cell parameters from 1351 reflections
*c* = 15.9604 (14) Å	θ = 8.9–35.3°
α = 90.019 (3)°	µ = 0.32 mm^−^^1^
β = 93.610 (3)°	*T* = 173 K
γ = 106.439 (3)°	Cube, colorless
*V* = 530.90 (8) Å^3^	0.15 × 0.13 × 0.09 mm

### 4-[(Methylsulfonyloxy)methyl]-2,4,4a,6,8,8a-hexahydro-[1,3]dioxino[5,4-d][1,3]dioxine (1) Data collection

**Table:**

Bruker D8 Quest diffractometer	2290 reflections with *I* > 2σ(*I*)
Kappa Diffractometer scans	*R*_int_ = 0.084
Absorption correction: multi-scan (SADABS; Krause *et al.*, 2015)	θ_max_ = 41.3°, θ_min_ = 2.6°
*T*_min_ = 0.665, *T*_max_ = 0.748	*h* = −8→7
15993 measured reflections	*k* = −11→11
4823 independent reflections	*l* = −25→21

### 4-[(Methylsulfonyloxy)methyl]-2,4,4a,6,8,8a-hexahydro-[1,3]dioxino[5,4-d][1,3]dioxine (1) Refinement

**Table:**

Refinement on *F*^2^	0 restraints
Least-squares matrix: full	Hydrogen site location: inferred from neighbouring sites
*R*[*F*^2^ > 2σ(*F*^2^)] = 0.070	H-atom parameters constrained
*wR*(*F*^2^) = 0.147	*w* = 1/[σ^2^(*F*_o_^2^) + (0.0418*P*)^2^ + 0.2188*P*] where *P* = (*F*_o_^2^ + 2*F*_c_^2^)/3
*S* = 1.01	(Δ/σ)_max_ < 0.001
4823 reflections	Δρ_max_ = 0.41 e Å^−^^3^
146 parameters	Δρ_min_ = −0.65 e Å^−^^3^

### 4-[(Methylsulfonyloxy)methyl]-2,4,4a,6,8,8a-hexahydro-[1,3]dioxino[5,4-d][1,3]dioxine (1) Special details

**Table:**

Geometry. All esds (except the esd in the dihedral angle between two l.s. planes) are estimated using the full covariance matrix. The cell esds are taken into account individually in the estimation of esds in distances, angles and torsion angles; correlations between esds in cell parameters are only used when they are defined by crystal symmetry. An approximate (isotropic) treatment of cell esds is used for estimating esds involving l.s. planes.

### 4-[(Methylsulfonyloxy)methyl]-2,4,4a,6,8,8a-hexahydro-[1,3]dioxino[5,4-d][1,3]dioxine (1) Fractional atomic coordinates and isotropic or equivalent isotropic displacement parameters (Å^2^)

**Table:**

	*x*	*y*	*z*	*U*_iso_*/*U*_eq_	
S1	0.60628 (13)	0.80009 (9)	0.58781 (4)	0.02721 (16)	
O1	0.6927 (3)	0.4016 (2)	0.93342 (9)	0.0266 (4)	
O3	0.6751 (3)	0.6081 (2)	0.82311 (9)	0.0260 (4)	
O5	0.4827 (3)	0.2098 (2)	0.76954 (9)	0.0243 (3)	
O7	0.4434 (3)	0.0034 (2)	0.88198 (10)	0.0289 (4)	
O8	0.7865 (3)	0.7367 (2)	0.66251 (9)	0.0261 (4)	
O9	0.3104 (4)	0.6800 (3)	0.58398 (11)	0.0398 (5)	
O10	0.6589 (4)	0.9992 (2)	0.60079 (10)	0.0373 (4)	
C2	0.7642 (6)	0.5925 (3)	0.90849 (14)	0.0309 (5)	
H2A	0.979758	0.650217	0.917189	0.037*	
H2B	0.666347	0.663938	0.943971	0.037*	
C4	0.8303 (5)	0.5179 (3)	0.77023 (13)	0.0227 (4)	
H4	1.045638	0.585886	0.776752	0.027*	
C4A	0.7829 (5)	0.3113 (3)	0.79322 (13)	0.0216 (4)	
H4A	0.917530	0.256771	0.761967	0.026*	
C6	0.4217 (6)	0.0183 (3)	0.79414 (14)	0.0304 (5)	
H6A	0.220286	−0.052390	0.772201	0.036*	
H6B	0.562188	−0.040402	0.769413	0.036*	
C8	0.7415 (5)	0.0902 (3)	0.91348 (14)	0.0275 (5)	
H8A	0.871338	0.019144	0.891627	0.033*	
H8B	0.754402	0.083849	0.975507	0.033*	
C8A	0.8461 (5)	0.2954 (3)	0.88766 (13)	0.0228 (4)	
H8AA	1.062871	0.346026	0.901998	0.027*	
C9	0.7183 (5)	0.5344 (3)	0.68109 (13)	0.0240 (5)	
H9A	0.815314	0.469652	0.642238	0.029*	
H9B	0.502868	0.474287	0.674387	0.029*	
C10	0.7686 (5)	0.7580 (3)	0.49760 (14)	0.0280 (5)	
H10A	0.666135	0.795460	0.448192	0.042*	
H10B	0.754631	0.622437	0.493002	0.042*	
H10C	0.976420	0.832549	0.500840	0.042*	

### 4-[(Methylsulfonyloxy)methyl]-2,4,4a,6,8,8a-hexahydro-[1,3]dioxino[5,4-d][1,3]dioxine (1) Atomic displacement parameters (Å^2^)

**Table:**

	*U*^11^	*U*^22^	*U*^33^	*U*^12^	*U*^13^	*U*^23^
S1	0.0286 (3)	0.0314 (3)	0.0257 (3)	0.0142 (2)	0.0054 (2)	0.0082 (2)
O1	0.0368 (9)	0.0221 (8)	0.0199 (8)	0.0062 (7)	0.0049 (7)	0.0028 (6)
O3	0.0357 (9)	0.0248 (8)	0.0203 (8)	0.0130 (7)	0.0034 (6)	0.0021 (6)
O5	0.0261 (8)	0.0208 (8)	0.0242 (8)	0.0038 (6)	0.0000 (6)	−0.0001 (6)
O7	0.0339 (9)	0.0239 (9)	0.0257 (8)	0.0023 (7)	0.0056 (7)	0.0034 (6)
O8	0.0320 (9)	0.0231 (8)	0.0226 (8)	0.0067 (7)	0.0026 (6)	0.0073 (6)
O9	0.0237 (9)	0.0517 (12)	0.0463 (11)	0.0140 (8)	0.0052 (8)	0.0153 (9)
O10	0.0571 (12)	0.0318 (10)	0.0316 (10)	0.0246 (9)	0.0116 (8)	0.0086 (7)
C2	0.0457 (15)	0.0230 (12)	0.0222 (11)	0.0073 (10)	0.0006 (10)	0.0011 (9)
C4	0.0237 (11)	0.0212 (11)	0.0228 (11)	0.0056 (8)	0.0026 (8)	0.0038 (8)
C4A	0.0222 (11)	0.0229 (11)	0.0214 (11)	0.0087 (8)	0.0046 (8)	0.0035 (8)
C6	0.0416 (14)	0.0206 (12)	0.0262 (12)	0.0042 (10)	0.0021 (10)	0.0012 (9)
C8	0.0339 (13)	0.0262 (12)	0.0236 (11)	0.0100 (10)	0.0039 (9)	0.0068 (9)
C8A	0.0233 (11)	0.0221 (11)	0.0223 (11)	0.0051 (9)	0.0015 (8)	0.0047 (8)
C9	0.0303 (12)	0.0194 (11)	0.0218 (11)	0.0060 (9)	0.0021 (9)	0.0042 (8)
C10	0.0310 (13)	0.0306 (13)	0.0240 (12)	0.0111 (10)	0.0026 (9)	0.0043 (9)

### 4-[(Methylsulfonyloxy)methyl]-2,4,4a,6,8,8a-hexahydro-[1,3]dioxino[5,4-d][1,3]dioxine (1) Geometric parameters (Å, º)

**Table:**

S1—O10	1.4228 (18)	C4—C4A	1.516 (3)
S1—O9	1.4270 (18)	C4—H4	1.0000
S1—O8	1.5698 (15)	C4A—C8A	1.529 (3)
S1—C10	1.742 (2)	C4A—H4A	1.0000
O1—C2	1.408 (3)	C6—H6A	0.9900
O1—C8A	1.434 (3)	C6—H6B	0.9900
O3—C2	1.415 (3)	C8—C8A	1.511 (3)
O3—C4	1.430 (3)	C8—H8A	0.9900
O5—C6	1.412 (3)	C8—H8B	0.9900
O5—C4A	1.433 (3)	C8A—H8AA	1.0000
O7—C6	1.406 (3)	C9—H9A	0.9900
O7—C8	1.434 (3)	C9—H9B	0.9900
O8—C9	1.461 (2)	C10—H10A	0.9800
C2—H2A	0.9900	C10—H10B	0.9800
C2—H2B	0.9900	C10—H10C	0.9800
C4—C9	1.504 (3)		
			
O10—S1—O9	119.11 (11)	O7—C6—H6A	109.3
O10—S1—O8	104.77 (10)	O5—C6—H6A	109.3
O9—S1—O8	108.86 (9)	O7—C6—H6B	109.3
O10—S1—C10	109.80 (11)	O5—C6—H6B	109.3
O9—S1—C10	108.16 (12)	H6A—C6—H6B	108.0
O8—S1—C10	105.28 (10)	O7—C8—C8A	111.14 (18)
C2—O1—C8A	111.28 (17)	O7—C8—H8A	109.4
C2—O3—C4	110.29 (17)	C8A—C8—H8A	109.4
C6—O5—C4A	110.55 (17)	O7—C8—H8B	109.4
C6—O7—C8	109.65 (17)	C8A—C8—H8B	109.4
C9—O8—S1	118.45 (13)	H8A—C8—H8B	108.0
O1—C2—O3	111.64 (18)	O1—C8A—C8	107.96 (18)
O1—C2—H2A	109.3	O1—C8A—C4A	110.20 (17)
O3—C2—H2A	109.3	C8—C8A—C4A	110.23 (18)
O1—C2—H2B	109.3	O1—C8A—H8AA	109.5
O3—C2—H2B	109.3	C8—C8A—H8AA	109.5
H2A—C2—H2B	108.0	C4A—C8A—H8AA	109.5
O3—C4—C9	107.43 (17)	O8—C9—C4	107.50 (17)
O3—C4—C4A	110.85 (17)	O8—C9—H9A	110.2
C9—C4—C4A	110.92 (18)	C4—C9—H9A	110.2
O3—C4—H4	109.2	O8—C9—H9B	110.2
C9—C4—H4	109.2	C4—C9—H9B	110.2
C4A—C4—H4	109.2	H9A—C9—H9B	108.5
O5—C4A—C4	108.19 (17)	S1—C10—H10A	109.5
O5—C4A—C8A	110.03 (17)	S1—C10—H10B	109.5
C4—C4A—C8A	110.15 (18)	H10A—C10—H10B	109.5
O5—C4A—H4A	109.5	S1—C10—H10C	109.5
C4—C4A—H4A	109.5	H10A—C10—H10C	109.5
C8A—C4A—H4A	109.5	H10B—C10—H10C	109.5
O7—C6—O5	111.57 (18)		
			
O10—S1—O8—C9	163.58 (16)	C4A—O5—C6—O7	64.7 (2)
O9—S1—O8—C9	35.15 (18)	C6—O7—C8—C8A	56.9 (2)
C10—S1—O8—C9	−80.62 (17)	C2—O1—C8A—C8	−176.03 (18)
C8A—O1—C2—O3	63.0 (2)	C2—O1—C8A—C4A	−55.6 (2)
C4—O3—C2—O1	−63.2 (2)	O7—C8—C8A—O1	70.2 (2)
C2—O3—C4—C9	178.21 (17)	O7—C8—C8A—C4A	−50.2 (2)
C2—O3—C4—C4A	56.9 (2)	O5—C4A—C8A—O1	−69.6 (2)
C6—O5—C4A—C4	−176.51 (17)	C4—C4A—C8A—O1	49.6 (2)
C6—O5—C4A—C8A	−56.1 (2)	O5—C4A—C8A—C8	49.5 (2)
O3—C4—C4A—O5	69.7 (2)	C4—C4A—C8A—C8	168.64 (18)
C9—C4—C4A—O5	−49.6 (2)	S1—O8—C9—C4	−159.17 (14)
O3—C4—C4A—C8A	−50.7 (2)	O3—C4—C9—O8	61.8 (2)
C9—C4—C4A—C8A	−169.91 (18)	C4A—C4—C9—O8	−176.93 (17)
C8—O7—C6—O5	−64.3 (2)		

### 4-[(Benzyloxy)methyl]-2,4,4a,6,8,8a-hexahydro-[1,3]dioxino[5,4-d][1,3]dioxine (2) Crystal data

**Table:**

C_14_H_18_O_5_	*F*(000) = 568
*M**_r_* = 266.28	*D*_x_ = 1.398 Mg m^−^^3^
Monoclinic, *P*2_1_/*c*	Mo *K*α radiation, λ = 0.71073 Å
*a* = 20.5429 (9) Å	Cell parameters from 665 reflections
*b* = 4.4574 (2) Å	θ = 7.0–30.6°
*c* = 13.9148 (7) Å	µ = 0.11 mm^−^^1^
β = 96.651 (2)°	*T* = 173 K
*V* = 1265.57 (10) Å^3^	Block, colorless
*Z* = 4	0.20 × 0.10 × 0.08 mm

### 4-[(Benzyloxy)methyl]-2,4,4a,6,8,8a-hexahydro-[1,3]dioxino[5,4-d][1,3]dioxine (2) Data collection

**Table:**

Bruker D8 Quest diffractometer	2759 reflections with *I* > 2σ(*I*)
Kappa Diffractometer scans	*R*_int_ = 0.062
Absorption correction: multi-scan (SADABS; Krause *et al.*, 2015)	θ_max_ = 30.6°, θ_min_ = 2.0°
*T*_min_ = 0.712, *T*_max_ = 0.746	*h* = −29→28
32635 measured reflections	*k* = −6→6
3878 independent reflections	*l* = −19→19

### 4-[(Benzyloxy)methyl]-2,4,4a,6,8,8a-hexahydro-[1,3]dioxino[5,4-d][1,3]dioxine (2) Refinement

**Table:**

Refinement on *F*^2^	0 restraints
Least-squares matrix: full	Hydrogen site location: inferred from neighbouring sites
*R*[*F*^2^ > 2σ(*F*^2^)] = 0.051	H-atom parameters constrained
*wR*(*F*^2^) = 0.123	*w* = 1/[σ^2^(*F*_o_^2^) + (0.0472*P*)^2^ + 0.495*P*] where *P* = (*F*_o_^2^ + 2*F*_c_^2^)/3
*S* = 1.04	(Δ/σ)_max_ < 0.001
3878 reflections	Δρ_max_ = 0.31 e Å^−^^3^
172 parameters	Δρ_min_ = −0.21 e Å^−^^3^

### 4-[(Benzyloxy)methyl]-2,4,4a,6,8,8a-hexahydro-[1,3]dioxino[5,4-d][1,3]dioxine (2) Special details

**Table:**

Geometry. All esds (except the esd in the dihedral angle between two l.s. planes) are estimated using the full covariance matrix. The cell esds are taken into account individually in the estimation of esds in distances, angles and torsion angles; correlations between esds in cell parameters are only used when they are defined by crystal symmetry. An approximate (isotropic) treatment of cell esds is used for estimating esds involving l.s. planes.

### 4-[(Benzyloxy)methyl]-2,4,4a,6,8,8a-hexahydro-[1,3]dioxino[5,4-d][1,3]dioxine (2) Fractional atomic coordinates and isotropic or equivalent isotropic displacement parameters (Å^2^)

**Table:**

	*x*	*y*	*z*	*U*_iso_*/*U*_eq_	
O3	0.14034 (5)	0.5880 (2)	0.31935 (7)	0.0230 (2)	
O5	0.18458 (5)	0.5729 (2)	0.12265 (7)	0.0205 (2)	
O7	0.09777 (5)	0.5175 (2)	0.00055 (7)	0.0244 (2)	
O8	0.27719 (5)	0.4412 (3)	0.40210 (7)	0.0277 (2)	
O1	0.05578 (5)	0.4848 (2)	0.19753 (7)	0.0223 (2)	
C2	0.07448 (7)	0.4955 (4)	0.29813 (10)	0.0250 (3)	
H2A	0.068982	0.294409	0.326277	0.030*	
H2B	0.045623	0.637039	0.328068	0.030*	
C13	0.37677 (9)	0.4540 (4)	0.69810 (12)	0.0369 (4)	
H13	0.360471	0.499754	0.757563	0.044*	
C4	0.18415 (7)	0.3773 (3)	0.28339 (10)	0.0202 (3)	
H4	0.181883	0.184159	0.319425	0.024*	
C14	0.43189 (9)	0.2806 (4)	0.69818 (12)	0.0362 (4)	
H14	0.453646	0.206011	0.757327	0.043*	
C15	0.45538 (10)	0.2158 (5)	0.61234 (14)	0.0488 (5)	
H15	0.493685	0.096685	0.611887	0.059*	
C16	0.42333 (9)	0.3238 (5)	0.52620 (13)	0.0405 (4)	
H16	0.439881	0.276927	0.467000	0.049*	
C4A	0.16574 (7)	0.3179 (3)	0.17607 (10)	0.0191 (3)	
H4A	0.190262	0.137749	0.157023	0.023*	
C6	0.16623 (7)	0.5295 (3)	0.02246 (10)	0.0247 (3)	
H6A	0.183747	0.696081	−0.013987	0.030*	
H6B	0.185645	0.340146	0.001883	0.030*	
C8	0.07206 (7)	0.2614 (3)	0.04578 (10)	0.0238 (3)	
H8A	0.087998	0.076190	0.016981	0.029*	
H8B	0.023629	0.263209	0.033288	0.029*	
C9	0.25236 (7)	0.5044 (3)	0.30451 (10)	0.0239 (3)	
H9A	0.281348	0.414262	0.260268	0.029*	
H9B	0.251289	0.724063	0.293854	0.029*	
C12	0.34453 (8)	0.5633 (4)	0.61206 (12)	0.0355 (4)	
H12	0.306381	0.683312	0.612870	0.043*	
C8A	0.09237 (7)	0.2591 (3)	0.15380 (10)	0.0204 (3)	
H8AA	0.081985	0.058431	0.180316	0.024*	
C10	0.33439 (8)	0.6145 (4)	0.43094 (12)	0.0334 (4)	
H10A	0.322121	0.827277	0.438450	0.040*	
H10B	0.364623	0.603076	0.380580	0.040*	
C11	0.36794 (7)	0.4977 (3)	0.52499 (11)	0.0263 (3)	

### 4-[(Benzyloxy)methyl]-2,4,4a,6,8,8a-hexahydro-[1,3]dioxino[5,4-d][1,3]dioxine (2) Atomic displacement parameters (Å^2^)

**Table:**

	*U*^11^	*U*^22^	*U*^33^	*U*^12^	*U*^13^	*U*^23^
O3	0.0214 (5)	0.0255 (5)	0.0220 (5)	0.0013 (4)	0.0020 (4)	−0.0051 (4)
O5	0.0249 (5)	0.0202 (5)	0.0162 (4)	−0.0022 (4)	0.0016 (4)	0.0004 (4)
O7	0.0285 (5)	0.0231 (5)	0.0204 (5)	−0.0008 (4)	−0.0027 (4)	0.0034 (4)
O8	0.0248 (5)	0.0345 (6)	0.0219 (5)	−0.0064 (4)	−0.0057 (4)	0.0041 (4)
O1	0.0213 (5)	0.0238 (5)	0.0216 (5)	0.0014 (4)	0.0010 (4)	0.0002 (4)
C2	0.0207 (7)	0.0325 (8)	0.0221 (7)	0.0003 (6)	0.0038 (5)	0.0011 (6)
C13	0.0387 (10)	0.0449 (10)	0.0272 (8)	−0.0007 (8)	0.0036 (7)	−0.0043 (7)
C4	0.0226 (7)	0.0190 (6)	0.0186 (6)	0.0027 (5)	0.0005 (5)	0.0001 (5)
C14	0.0366 (9)	0.0401 (10)	0.0289 (8)	−0.0014 (7)	−0.0090 (7)	0.0027 (7)
C15	0.0406 (11)	0.0617 (13)	0.0427 (11)	0.0242 (10)	−0.0014 (8)	−0.0011 (9)
C16	0.0391 (10)	0.0533 (11)	0.0292 (9)	0.0116 (8)	0.0045 (7)	−0.0039 (8)
C4A	0.0234 (7)	0.0148 (6)	0.0187 (6)	0.0024 (5)	0.0007 (5)	0.0008 (5)
C6	0.0291 (8)	0.0281 (7)	0.0171 (6)	0.0003 (6)	0.0032 (5)	0.0005 (6)
C8	0.0281 (7)	0.0188 (6)	0.0231 (7)	−0.0016 (6)	−0.0037 (6)	−0.0013 (5)
C9	0.0233 (7)	0.0283 (7)	0.0194 (6)	0.0009 (6)	−0.0005 (5)	0.0019 (6)
C12	0.0265 (8)	0.0430 (10)	0.0365 (9)	0.0068 (7)	0.0020 (7)	−0.0028 (8)
C8A	0.0252 (7)	0.0149 (6)	0.0203 (6)	−0.0014 (5)	−0.0010 (5)	0.0012 (5)
C10	0.0286 (8)	0.0382 (9)	0.0310 (8)	−0.0100 (7)	−0.0071 (6)	0.0060 (7)
C11	0.0217 (7)	0.0294 (8)	0.0265 (7)	−0.0058 (6)	−0.0031 (6)	−0.0012 (6)

### 4-[(Benzyloxy)methyl]-2,4,4a,6,8,8a-hexahydro-[1,3]dioxino[5,4-d][1,3]dioxine (2) Geometric parameters (Å, º)

**Table:**

O3—C2	1.4125 (17)	C15—C16	1.385 (3)
O3—C4	1.4307 (17)	C15—H15	0.9500
O5—C6	1.4146 (16)	C16—C11	1.375 (2)
O5—C4A	1.4354 (16)	C16—H16	0.9500
O7—C6	1.4051 (18)	C4A—C8A	1.526 (2)
O7—C8	1.4336 (17)	C4A—H4A	1.0000
O8—C9	1.4223 (16)	C6—H6A	0.9900
O8—C10	1.4247 (18)	C6—H6B	0.9900
O1—C2	1.4089 (17)	C8—C8A	1.5127 (19)
O1—C8A	1.4329 (17)	C8—H8A	0.9900
C2—H2A	0.9900	C8—H8B	0.9900
C2—H2B	0.9900	C9—H9A	0.9900
C13—C14	1.371 (3)	C9—H9B	0.9900
C13—C12	1.388 (2)	C12—C11	1.385 (2)
C13—H13	0.9500	C12—H12	0.9500
C4—C9	1.509 (2)	C8A—H8AA	1.0000
C4—C4A	1.5206 (19)	C10—C11	1.500 (2)
C4—H4	1.0000	C10—H10A	0.9900
C14—C15	1.370 (3)	C10—H10B	0.9900
C14—H14	0.9500		
			
C2—O3—C4	111.19 (11)	O7—C6—H6A	109.3
C6—O5—C4A	110.19 (10)	O5—C6—H6A	109.3
C6—O7—C8	110.21 (11)	O7—C6—H6B	109.3
C9—O8—C10	110.75 (11)	O5—C6—H6B	109.3
C2—O1—C8A	110.46 (11)	H6A—C6—H6B	108.0
O1—C2—O3	111.22 (11)	O7—C8—C8A	111.62 (11)
O1—C2—H2A	109.4	O7—C8—H8A	109.3
O3—C2—H2A	109.4	C8A—C8—H8A	109.3
O1—C2—H2B	109.4	O7—C8—H8B	109.3
O3—C2—H2B	109.4	C8A—C8—H8B	109.3
H2A—C2—H2B	108.0	H8A—C8—H8B	108.0
C14—C13—C12	120.68 (16)	O8—C9—C4	109.50 (11)
C14—C13—H13	119.7	O8—C9—H9A	109.8
C12—C13—H13	119.7	C4—C9—H9A	109.8
O3—C4—C9	107.03 (11)	O8—C9—H9B	109.8
O3—C4—C4A	111.27 (11)	C4—C9—H9B	109.8
C9—C4—C4A	112.12 (12)	H9A—C9—H9B	108.2
O3—C4—H4	108.8	C11—C12—C13	120.07 (16)
C9—C4—H4	108.8	C11—C12—H12	120.0
C4A—C4—H4	108.8	C13—C12—H12	120.0
C15—C14—C13	119.51 (16)	O1—C8A—C8	108.61 (11)
C15—C14—H14	120.2	O1—C8A—C4A	110.34 (11)
C13—C14—H14	120.2	C8—C8A—C4A	110.66 (12)
C14—C15—C16	120.08 (17)	O1—C8A—H8AA	109.1
C14—C15—H15	120.0	C8—C8A—H8AA	109.1
C16—C15—H15	120.0	C4A—C8A—H8AA	109.1
C11—C16—C15	121.06 (16)	O8—C10—C11	109.77 (13)
C11—C16—H16	119.5	O8—C10—H10A	109.7
C15—C16—H16	119.5	C11—C10—H10A	109.7
O5—C4A—C4	108.63 (11)	O8—C10—H10B	109.7
O5—C4A—C8A	110.50 (11)	C11—C10—H10B	109.7
C4—C4A—C8A	110.90 (11)	H10A—C10—H10B	108.2
O5—C4A—H4A	108.9	C16—C11—C12	118.60 (15)
C4—C4A—H4A	108.9	C16—C11—C10	120.14 (15)
C8A—C4A—H4A	108.9	C12—C11—C10	121.26 (15)
O7—C6—O5	111.39 (11)		
			
C8A—O1—C2—O3	64.95 (15)	C4A—C4—C9—O8	−156.09 (11)
C4—O3—C2—O1	−63.31 (15)	C14—C13—C12—C11	0.0 (3)
C2—O3—C4—C9	176.82 (11)	C2—O1—C8A—C8	−178.47 (11)
C2—O3—C4—C4A	54.02 (14)	C2—O1—C8A—C4A	−57.01 (14)
C12—C13—C14—C15	0.2 (3)	O7—C8—C8A—O1	73.36 (15)
C13—C14—C15—C16	−0.3 (3)	O7—C8—C8A—C4A	−47.90 (15)
C14—C15—C16—C11	0.3 (3)	O5—C4A—C8A—O1	−71.99 (13)
C6—O5—C4A—C4	−178.11 (11)	C4—C4A—C8A—O1	48.53 (14)
C6—O5—C4A—C8A	−56.24 (14)	O5—C4A—C8A—C8	48.25 (14)
O3—C4—C4A—O5	74.48 (14)	C4—C4A—C8A—C8	168.77 (11)
C9—C4—C4A—O5	−45.35 (15)	C9—O8—C10—C11	−167.65 (13)
O3—C4—C4A—C8A	−47.14 (15)	C15—C16—C11—C12	−0.1 (3)
C9—C4—C4A—C8A	−166.97 (11)	C15—C16—C11—C10	179.44 (19)
C8—O7—C6—O5	−64.20 (15)	C13—C12—C11—C16	−0.1 (3)
C4A—O5—C6—O7	65.18 (14)	C13—C12—C11—C10	−179.57 (16)
C6—O7—C8—C8A	55.30 (15)	O8—C10—C11—C16	102.95 (19)
C10—O8—C9—C4	−166.65 (13)	O8—C10—C11—C12	−77.6 (2)
O3—C4—C9—O8	81.64 (14)		

### 4-[(Anilinocarbonyl)methyl]-2,4,4a,6,8,8a-hexahydro-[1,3]dioxino[5,4-d][1,3]dioxine (3) Crystal data

**Table:**

C_14_H_17_NO_6_	*F*(000) = 624
*M**_r_* = 295.28	*D*_x_ = 1.476 Mg m^−^^3^
Monoclinic, *P*2_1_/*c*	Mo *K*α radiation, λ = 0.71073 Å
*a* = 22.909 (2) Å	Cell parameters from 2795 reflections
*b* = 4.8973 (5) Å	θ = 7.0–41.7°
*c* = 12.2331 (14) Å	µ = 0.12 mm^−^^1^
β = 104.529 (4)°	*T* = 173 K
*V* = 1328.6 (2) Å^3^	Block, colorless
*Z* = 4	0.20 × 0.15 × 0.12 mm

### 4-[(Anilinocarbonyl)methyl]-2,4,4a,6,8,8a-hexahydro-[1,3]dioxino[5,4-d][1,3]dioxine (3) Data collection

**Table:**

Bruker D8 Venture Duo diffractometer	2803 reflections with *I* > 2σ(*I*)
Kappa Diffractometer scans	*R*_int_ = 0.069
Absorption correction: multi-scan (SADABS; Krause *et al.*, 2015)	θ_max_ = 27.5°, θ_min_ = 2.8°
*T*_min_ = 0.568, *T*_max_ = 0.746	*h* = −29→29
23485 measured reflections	*k* = −6→6
3040 independent reflections	*l* = −15→15

### 4-[(Anilinocarbonyl)methyl]-2,4,4a,6,8,8a-hexahydro-[1,3]dioxino[5,4-d][1,3]dioxine (3) Refinement

**Table:**

Refinement on *F*^2^	0 restraints
Least-squares matrix: full	Hydrogen site location: mixed
*R*[*F*^2^ > 2σ(*F*^2^)] = 0.044	H atoms treated by a mixture of independent and constrained refinement
*wR*(*F*^2^) = 0.107	*w* = 1/[σ^2^(*F*_o_^2^) + (0.0495*P*)^2^ + 0.442*P*] where *P* = (*F*_o_^2^ + 2*F*_c_^2^)/3
*S* = 1.06	(Δ/σ)_max_ = 0.001
3040 reflections	Δρ_max_ = 0.34 e Å^−^^3^
193 parameters	Δρ_min_ = −0.29 e Å^−^^3^

### 4-[(Anilinocarbonyl)methyl]-2,4,4a,6,8,8a-hexahydro-[1,3]dioxino[5,4-d][1,3]dioxine (3) Special details

**Table:**

Geometry. All esds (except the esd in the dihedral angle between two l.s. planes) are estimated using the full covariance matrix. The cell esds are taken into account individually in the estimation of esds in distances, angles and torsion angles; correlations between esds in cell parameters are only used when they are defined by crystal symmetry. An approximate (isotropic) treatment of cell esds is used for estimating esds involving l.s. planes.

### 4-[(Anilinocarbonyl)methyl]-2,4,4a,6,8,8a-hexahydro-[1,3]dioxino[5,4-d][1,3]dioxine (3) Fractional atomic coordinates and isotropic or equivalent isotropic displacement parameters (Å^2^)

**Table:**

	*x*	*y*	*z*	*U*_iso_*/*U*_eq_	
O1	0.60997 (4)	0.50342 (19)	0.58605 (7)	0.0310 (2)	
O3	0.70830 (4)	0.62699 (19)	0.58784 (7)	0.0302 (2)	
O5	0.63244 (4)	0.55251 (17)	0.35740 (7)	0.0258 (2)	
O7	0.53139 (4)	0.48658 (19)	0.35638 (8)	0.0324 (2)	
O8	0.77058 (3)	0.32337 (15)	0.37519 (7)	0.02338 (19)	
O9	0.82960 (4)	0.63477 (16)	0.31909 (8)	0.0330 (2)	
N1	0.82836 (4)	0.19036 (18)	0.26368 (8)	0.0225 (2)	
H1	0.8209 (7)	0.027 (3)	0.2824 (13)	0.027*	
C2	0.66883 (6)	0.5438 (3)	0.65284 (10)	0.0347 (3)	
H2A	0.683838	0.371609	0.692421	0.042*	
H2B	0.668196	0.684296	0.710706	0.042*	
C4	0.71453 (5)	0.4155 (2)	0.51082 (9)	0.0230 (2)	
H4	0.733167	0.251744	0.554844	0.028*	
C4A	0.65326 (5)	0.3365 (2)	0.43578 (9)	0.0215 (2)	
H4A	0.657592	0.166324	0.393308	0.026*	
C6	0.57349 (5)	0.4990 (3)	0.29052 (10)	0.0320 (3)	
H6A	0.561531	0.644689	0.233139	0.038*	
H6B	0.573382	0.323456	0.250366	0.038*	
C8	0.54503 (5)	0.2626 (3)	0.43325 (11)	0.0318 (3)	
H8A	0.540811	0.089413	0.390230	0.038*	
H8B	0.515926	0.259346	0.480937	0.038*	
C9	0.75822 (5)	0.5321 (2)	0.44922 (10)	0.0242 (2)	
H9A	0.740477	0.694399	0.404957	0.029*	
H9B	0.795986	0.587944	0.503863	0.029*	
C8A	0.60852 (5)	0.2844 (2)	0.50798 (10)	0.0262 (2)	
H8AA	0.619336	0.109986	0.550758	0.031*	
C10	0.81139 (5)	0.4021 (2)	0.31843 (9)	0.0208 (2)	
C11	0.87120 (5)	0.2165 (2)	0.19801 (9)	0.0210 (2)	
C12	0.86405 (5)	0.4148 (2)	0.11452 (10)	0.0265 (2)	
H12	0.831260	0.539301	0.102868	0.032*	
C13	0.90499 (6)	0.4305 (3)	0.04823 (10)	0.0310 (3)	
H13	0.900295	0.566736	−0.008600	0.037*	
C14	0.95263 (6)	0.2486 (3)	0.06448 (11)	0.0333 (3)	
H14	0.980372	0.258761	0.018488	0.040*	
C15	0.95972 (6)	0.0516 (3)	0.14805 (12)	0.0323 (3)	
H15	0.992606	−0.072340	0.159664	0.039*	
C16	0.91903 (5)	0.0342 (2)	0.21495 (10)	0.0256 (2)	
H16	0.923900	−0.101674	0.271959	0.031*	

### 4-[(Anilinocarbonyl)methyl]-2,4,4a,6,8,8a-hexahydro-[1,3]dioxino[5,4-d][1,3]dioxine (3) Atomic displacement parameters (Å^2^)

**Table:**

	*U*^11^	*U*^22^	*U*^33^	*U*^12^	*U*^13^	*U*^23^
O1	0.0299 (4)	0.0412 (5)	0.0242 (4)	0.0067 (4)	0.0111 (3)	−0.0022 (3)
O3	0.0292 (4)	0.0361 (5)	0.0248 (4)	0.0022 (3)	0.0056 (3)	−0.0100 (3)
O5	0.0216 (4)	0.0324 (4)	0.0222 (4)	0.0004 (3)	0.0032 (3)	0.0065 (3)
O7	0.0209 (4)	0.0425 (5)	0.0336 (5)	0.0030 (3)	0.0064 (3)	0.0023 (4)
O8	0.0225 (4)	0.0208 (4)	0.0287 (4)	−0.0030 (3)	0.0100 (3)	−0.0040 (3)
O9	0.0438 (5)	0.0157 (4)	0.0454 (5)	−0.0033 (3)	0.0221 (4)	−0.0004 (3)
N1	0.0248 (4)	0.0156 (4)	0.0291 (5)	−0.0020 (3)	0.0107 (4)	0.0010 (3)
C2	0.0344 (7)	0.0503 (8)	0.0194 (5)	0.0081 (6)	0.0068 (5)	−0.0035 (5)
C4	0.0227 (5)	0.0246 (5)	0.0211 (5)	0.0042 (4)	0.0043 (4)	−0.0016 (4)
C4A	0.0220 (5)	0.0225 (5)	0.0206 (5)	0.0023 (4)	0.0066 (4)	0.0006 (4)
C6	0.0220 (6)	0.0482 (7)	0.0241 (6)	0.0007 (5)	0.0025 (4)	0.0031 (5)
C8	0.0256 (6)	0.0345 (6)	0.0381 (6)	−0.0027 (5)	0.0135 (5)	−0.0003 (5)
C9	0.0217 (5)	0.0224 (5)	0.0284 (5)	0.0003 (4)	0.0064 (4)	−0.0052 (4)
C8A	0.0280 (6)	0.0273 (5)	0.0258 (5)	0.0031 (4)	0.0115 (4)	0.0037 (4)
C10	0.0196 (5)	0.0179 (5)	0.0240 (5)	0.0004 (4)	0.0040 (4)	0.0025 (4)
C11	0.0220 (5)	0.0188 (5)	0.0222 (5)	−0.0040 (4)	0.0057 (4)	−0.0025 (4)
C12	0.0291 (6)	0.0230 (5)	0.0263 (5)	−0.0026 (4)	0.0046 (4)	0.0020 (4)
C13	0.0417 (7)	0.0277 (6)	0.0242 (5)	−0.0089 (5)	0.0093 (5)	0.0015 (4)
C14	0.0406 (7)	0.0318 (6)	0.0336 (6)	−0.0095 (5)	0.0204 (5)	−0.0064 (5)
C15	0.0316 (6)	0.0275 (6)	0.0418 (7)	0.0002 (5)	0.0165 (5)	−0.0037 (5)
C16	0.0279 (6)	0.0209 (5)	0.0288 (6)	−0.0005 (4)	0.0086 (4)	0.0006 (4)

### 4-[(Anilinocarbonyl)methyl]-2,4,4a,6,8,8a-hexahydro-[1,3]dioxino[5,4-d][1,3]dioxine (3) Geometric parameters (Å, º)

**Table:**

O1—C2	1.4042 (16)	C4A—H4A	1.0000
O1—C8A	1.4309 (14)	C6—H6A	0.9900
O3—C2	1.4059 (16)	C6—H6B	0.9900
O3—C4	1.4313 (13)	C8—C8A	1.5152 (17)
O5—C6	1.4168 (14)	C8—H8A	0.9900
O5—C4A	1.4272 (13)	C8—H8B	0.9900
O7—C6	1.4046 (15)	C9—H9A	0.9900
O7—C8	1.4275 (16)	C9—H9B	0.9900
O8—C10	1.3530 (13)	C8A—H8AA	1.0000
O8—C9	1.4400 (13)	C11—C16	1.3881 (16)
O9—C10	1.2129 (13)	C11—C12	1.3891 (15)
N1—C10	1.3436 (14)	C12—C13	1.3873 (17)
N1—C11	1.4215 (14)	C12—H12	0.9500
N1—H1	0.863 (16)	C13—C14	1.384 (2)
C2—H2A	0.9900	C13—H13	0.9500
C2—H2B	0.9900	C14—C15	1.3852 (18)
C4—C9	1.5079 (15)	C14—H14	0.9500
C4—C4A	1.5219 (15)	C15—C16	1.3888 (16)
C4—H4	1.0000	C15—H15	0.9500
C4A—C8A	1.5330 (15)	C16—H16	0.9500
			
C2—O1—C8A	110.61 (9)	C8A—C8—H8B	109.4
C2—O3—C4	110.31 (9)	H8A—C8—H8B	108.0
C6—O5—C4A	111.13 (9)	O8—C9—C4	107.79 (9)
C6—O7—C8	110.06 (9)	O8—C9—H9A	110.1
C10—O8—C9	113.00 (8)	C4—C9—H9A	110.1
C10—N1—C11	122.64 (9)	O8—C9—H9B	110.1
C10—N1—H1	118.9 (10)	C4—C9—H9B	110.1
C11—N1—H1	116.4 (10)	H9A—C9—H9B	108.5
O1—C2—O3	111.82 (9)	O1—C8A—C8	108.28 (9)
O1—C2—H2A	109.3	O1—C8A—C4A	110.67 (9)
O3—C2—H2A	109.3	C8—C8A—C4A	110.10 (9)
O1—C2—H2B	109.3	O1—C8A—H8AA	109.3
O3—C2—H2B	109.3	C8—C8A—H8AA	109.3
H2A—C2—H2B	107.9	C4A—C8A—H8AA	109.3
O3—C4—C9	104.08 (9)	O9—C10—N1	125.90 (10)
O3—C4—C4A	110.60 (8)	O9—C10—O8	123.03 (10)
C9—C4—C4A	114.97 (9)	N1—C10—O8	111.08 (9)
O3—C4—H4	109.0	C16—C11—C12	120.17 (10)
C9—C4—H4	109.0	C16—C11—N1	119.26 (10)
C4A—C4—H4	109.0	C12—C11—N1	120.52 (10)
O5—C4A—C4	108.61 (9)	C13—C12—C11	119.75 (11)
O5—C4A—C8A	110.56 (8)	C13—C12—H12	120.1
C4—C4A—C8A	110.09 (9)	C11—C12—H12	120.1
O5—C4A—H4A	109.2	C14—C13—C12	120.31 (11)
C4—C4A—H4A	109.2	C14—C13—H13	119.8
C8A—C4A—H4A	109.2	C12—C13—H13	119.8
O7—C6—O5	111.66 (9)	C13—C14—C15	119.77 (11)
O7—C6—H6A	109.3	C13—C14—H14	120.1
O5—C6—H6A	109.3	C15—C14—H14	120.1
O7—C6—H6B	109.3	C14—C15—C16	120.39 (11)
O5—C6—H6B	109.3	C14—C15—H15	119.8
H6A—C6—H6B	108.0	C16—C15—H15	119.8
O7—C8—C8A	111.20 (10)	C11—C16—C15	119.60 (11)
O7—C8—H8A	109.4	C11—C16—H16	120.2
C8A—C8—H8A	109.4	C15—C16—H16	120.2
O7—C8—H8B	109.4		
			
C8A—O1—C2—O3	64.04 (13)	O7—C8—C8A—C4A	−49.89 (13)
C4—O3—C2—O1	−64.56 (13)	O5—C4A—C8A—O1	−71.14 (11)
C2—O3—C4—C9	−179.25 (9)	C4—C4A—C8A—O1	48.87 (12)
C2—O3—C4—C4A	56.74 (11)	O5—C4A—C8A—C8	48.53 (12)
C6—O5—C4A—C4	−175.73 (9)	C4—C4A—C8A—C8	168.54 (9)
C6—O5—C4A—C8A	−54.84 (11)	C11—N1—C10—O9	0.04 (18)
O3—C4—C4A—O5	71.73 (11)	C11—N1—C10—O8	−179.59 (9)
C9—C4—C4A—O5	−45.78 (12)	C9—O8—C10—O9	−8.87 (15)
O3—C4—C4A—C8A	−49.46 (12)	C9—O8—C10—N1	170.76 (9)
C9—C4—C4A—C8A	−166.96 (9)	C10—N1—C11—C16	130.24 (11)
C8—O7—C6—O5	−63.89 (13)	C10—N1—C11—C12	−52.41 (15)
C4A—O5—C6—O7	63.36 (13)	C16—C11—C12—C13	−0.13 (16)
C6—O7—C8—C8A	57.21 (13)	N1—C11—C12—C13	−177.46 (10)
C10—O8—C9—C4	−177.55 (8)	C11—C12—C13—C14	0.38 (17)
O3—C4—C9—O8	175.86 (8)	C12—C13—C14—C15	−0.58 (19)
C4A—C4—C9—O8	−63.01 (12)	C13—C14—C15—C16	0.53 (19)
C2—O1—C8A—C8	−176.28 (10)	C12—C11—C16—C15	0.09 (17)
C2—O1—C8A—C4A	−55.53 (12)	N1—C11—C16—C15	177.45 (10)
O7—C8—C8A—O1	71.22 (12)	C14—C15—C16—C11	−0.29 (18)

### 4-[(Anilinocarbonyl)methyl]-2,4,4a,6,8,8a-hexahydro-[1,3]dioxino[5,4-d][1,3]dioxine (3) Hydrogen-bond geometry (Å, º)

**Table:**

*D*—H···*A*	*D*—H	H···*A*	*D*···*A*	*D*—H···*A*
N1—H1···O9^i^	0.863 (16)	1.969 (16)	2.8025 (13)	161.9 (14)
C2—H2*B*···O5^ii^	0.99	2.51	3.4515 (15)	159

Symmetry codes: (i) *x*, *y*−1, *z*; (ii) *x*, −*y*+3/2, *z*+1/2.
