# Supplementary material for: Structures of rac-2,4:3,5-di­methyl­ene xylitol ­derivatives
Source: Acta Crystallogr E Crystallogr Commun. 2023 Aug 4;79(Pt 9):786–90. doi: 10.1107/S2056989023006497 (PMC10483560; doi:10.1107/S2056989023006497)

## Structures of rac-2,4;3,5-dimethylene xylitol derivatives


## Michael Satlow and Paul G. Williard


## NMR spectra

All $^{1}H$ and $^{13}C$ NMR spectra for compounds **1-3** were acquired on a Bruker spectrometer with an Advance console at approx. 400 MHz and 100 MHz respectively and were processed with Bruker Topspin® software. Solvents utilized were $CDCl_3$ for compounds **1** and **2** and DMSO-$d_6$ for compound **3**. Chemical shifts are referenced relative to the respective solvents. Chemical structures are depicted on each spectrum that are provided in the following order.

Compound 1 – $^{1}H$ NMR
Compound 1 – $^{13}C$ NMR
Compound 2 – $^{1}H$ NMR
Compound 2 – $^{13}C$ NMR
Compound 3 – $^{1}H$ NMR
Compound 3 – $^{13}C$ NMR

2022-07-06-MesylateEster-white 1 1 /opt/topspin/data/ms

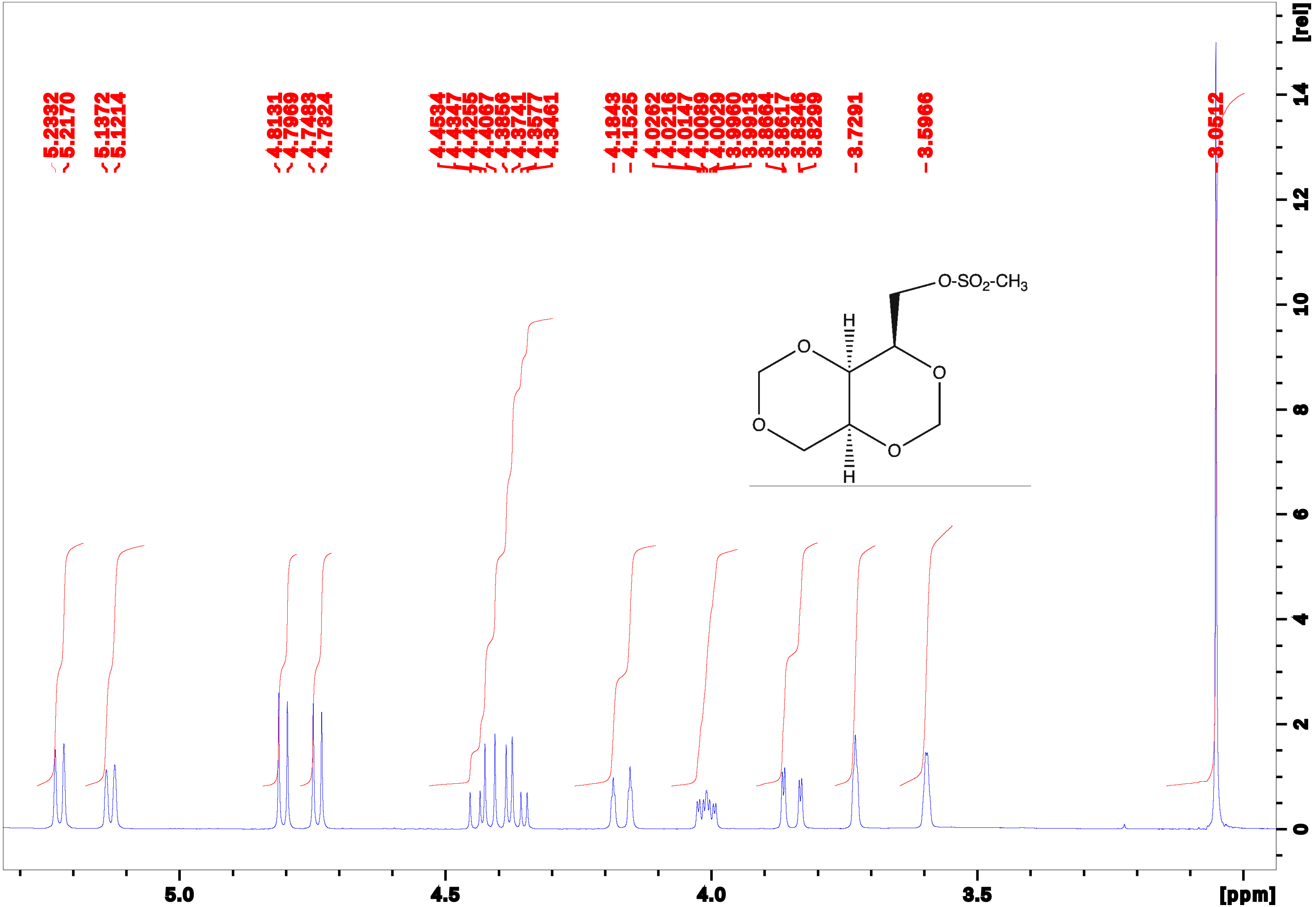

2022-07-06-MesylateEster-white 2 1 /opt/topspin/data/ms

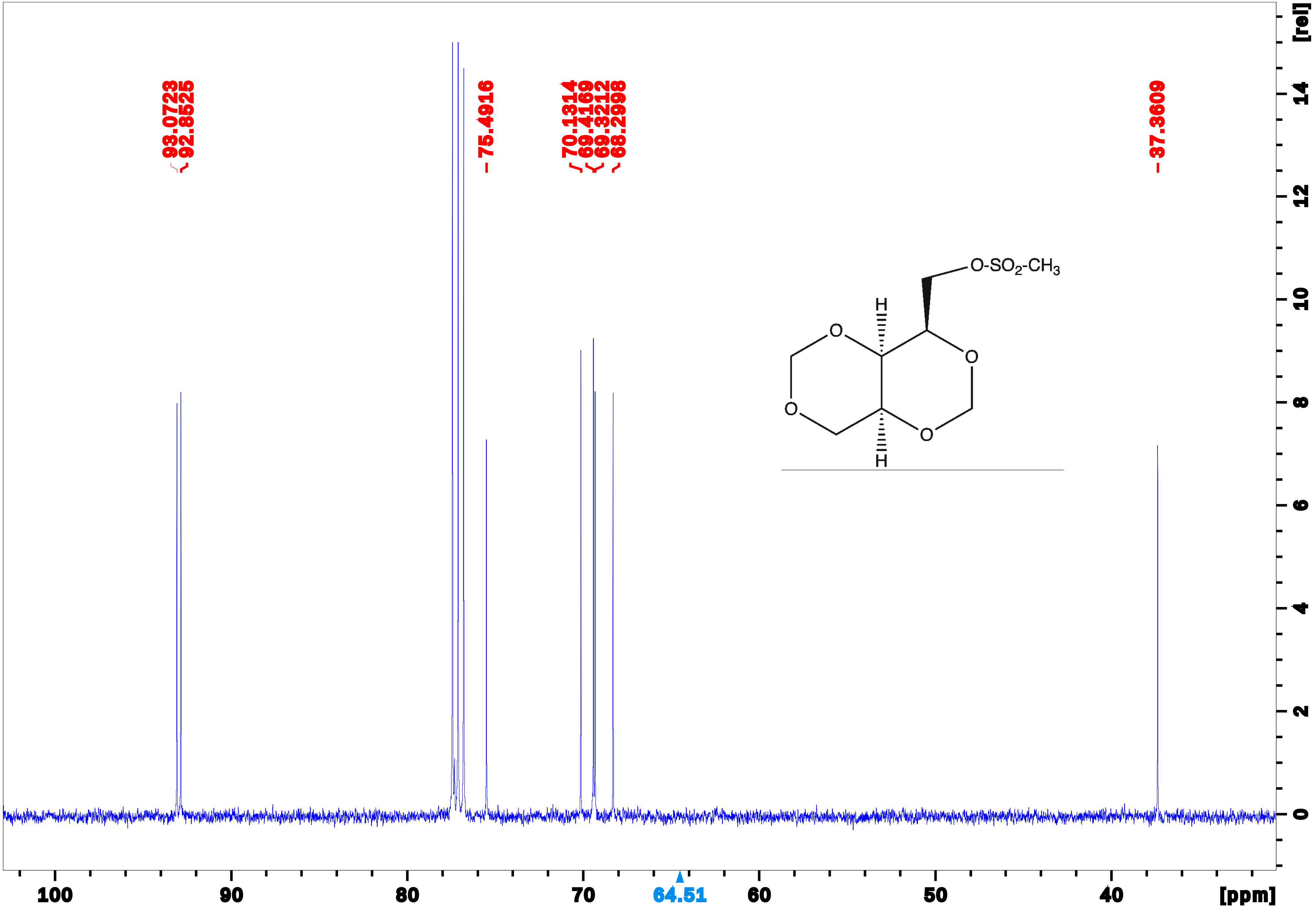

25aug22_xylitol-O-Bn-diethylidene 2 1 /opt/topspin/data/ms

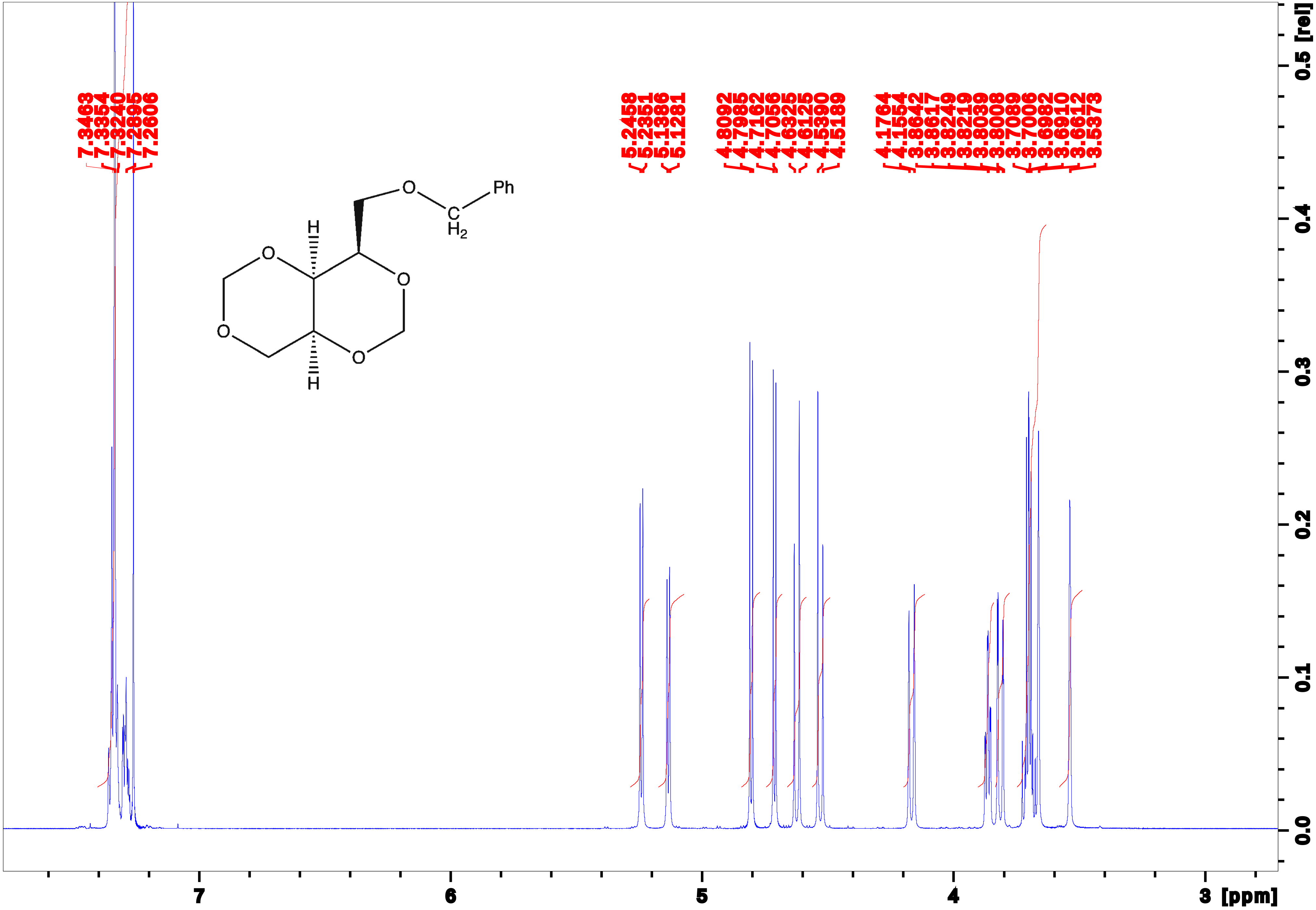

25aug22_xylitol-O-Bn-diethylidene 1 1 /opt/topspin/data/ms

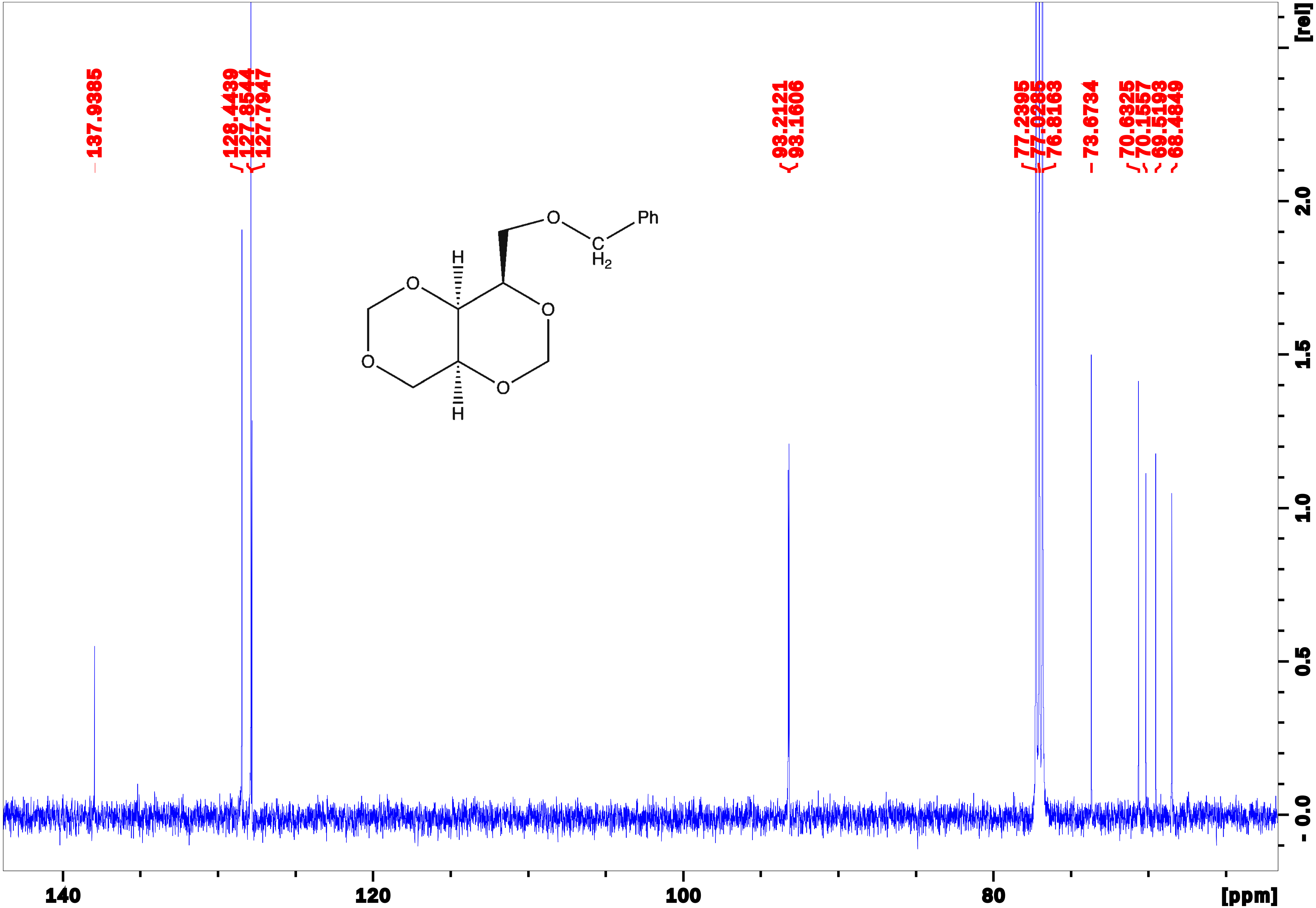

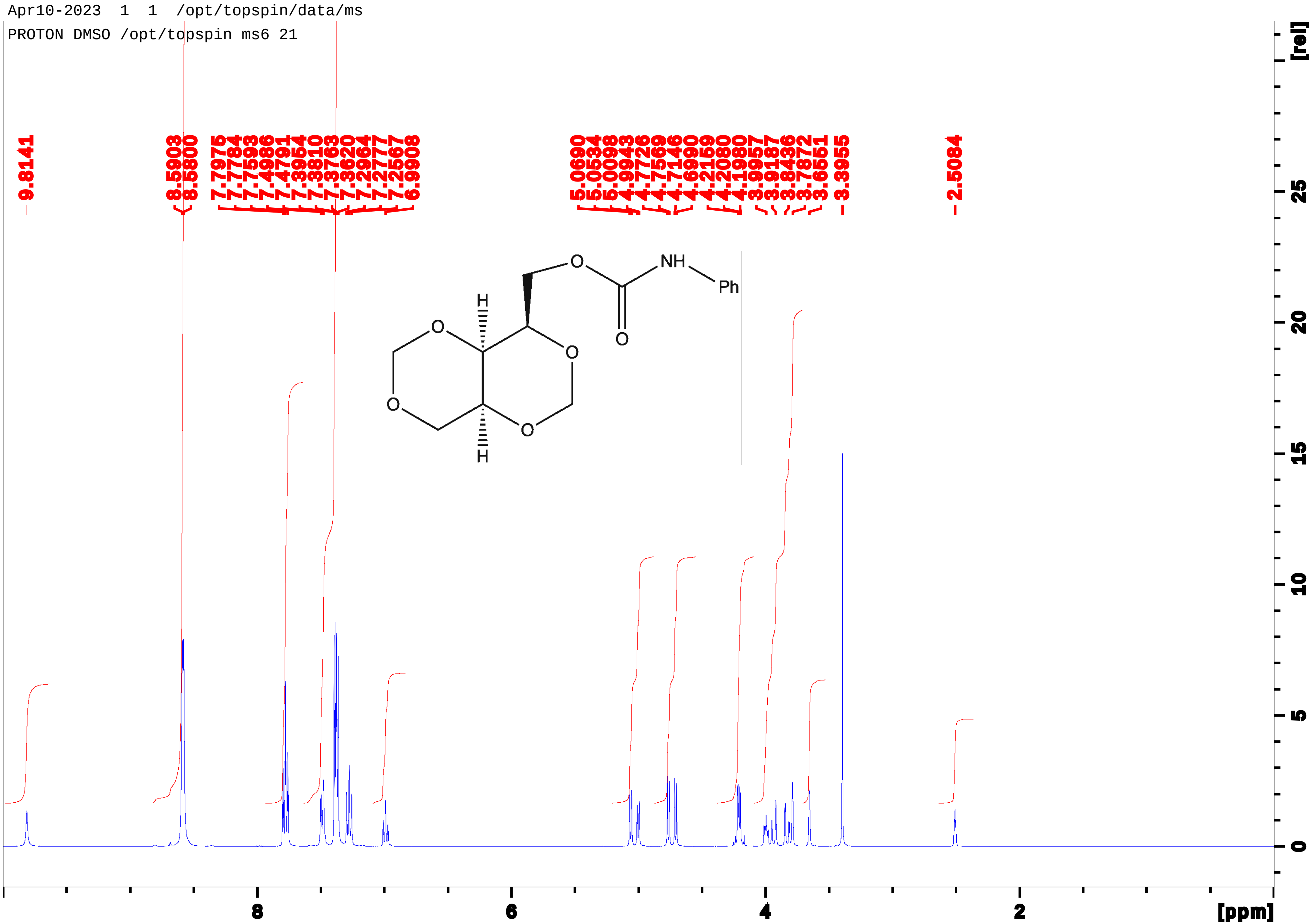
Apr10-2023 1 1 /opt/topspin/data/ms
PROTON DMSO /opt/topspin ms6 21
9.8141
8.5903
8.5800
7.7975
7.7784
7.7593
7.4986
7.4791
7.3954
7.3810
7.3763
7.3620
7.2964
7.2777
7.2567
6.9908
5.0690
5.0534
5.0098
4.9943
4.7726
4.7569
4.7146
4.6990
4.2159
4.2080
4.1980
3.9957
3.9187
3.8436
3.7872
3.6551
3.3955
2.5084
H
O
O
O
O
H
O
NH
Ph
O
[rel]
25
20
15
10
5
0
8
6
4
2
[ppm]

Apr10-2023  2  1  /opt/topspin/data/ms

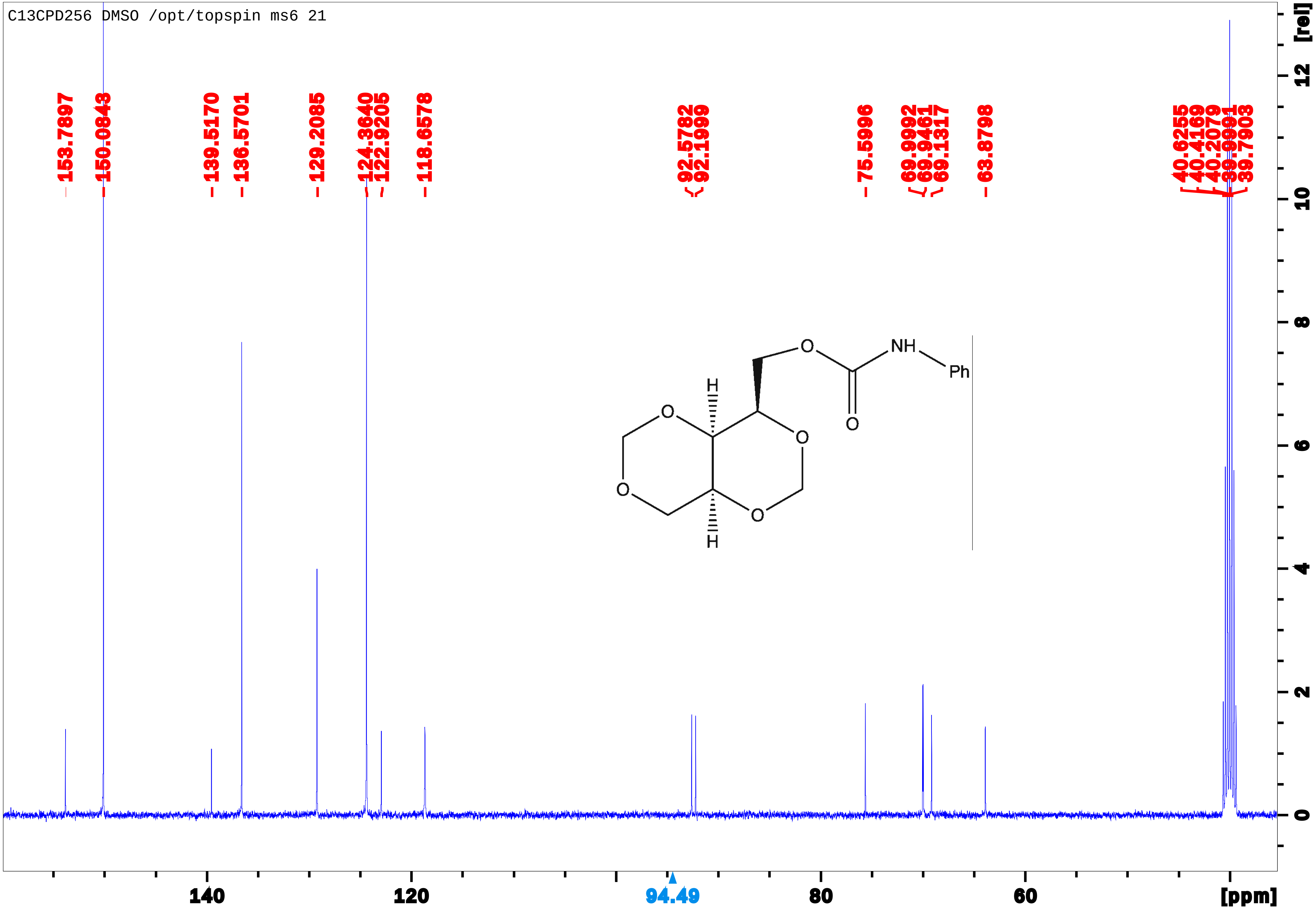

Supplement: Supplementary file 8 [file e-79-00786-sup8.pdf]
